# The Role of Diagnostic Laparoscopy in Staging and Treatment Planning of Gastric Cancer: A 400-Patient Retrospective Cohort Study

**DOI:** 10.3390/diagnostics16162590

**Published:** 2026-08-16

**Authors:** Tugce Kubra Gunes Capar, Pırıltı Ozcan, Muzeyyen Muge Savas, Ozgul Duzgun, Melike Ozcelik

**Affiliations:** 1 Division of Medical Oncology, Department of Internal Medicine, University of Health Sciences, Istanbul Umraniye Training and Research Hospital, Istanbul 34764, Türkiye; drmelike.ozcelik@gmail.com; 2 Department of General Surgery, Faculty of Medicine, Biruni University, Istanbul 34015, Türkiye; piriltiozcan@gmail.com; 3 Department of Pathology, University of Health Sciences, Istanbul Umraniye Training and Research Hospital, Istanbul 34764, Türkiye; mzyyn.p@hotmail.com; 4 Department of Surgical Oncology, University of Health Sciences, Istanbul Umraniye Training and Research Hospital, Istanbul 34764, Türkiye; ozgulduzgun@gmail.com

**Keywords:** gastric cancer, diagnostic laparoscopy, peritoneal carcinomatosis, HIPEC, PIPAC, PCI score

## Abstract

**Background**: Accurate staging in gastric cancer is essential for optimal treatment planning. Diagnostic laparoscopy (DL) plays a crucial role in detecting occult peritoneal metastases not identified by conventional imaging. This study aimed to evaluate the impact of DL on clinical decision-making and treatment allocation in patients with gastric cancer. **Methods**: We retrospectively analyzed consecutive patients treated between June 2016 and March 2026 at a tertiary Surgical Oncology Center. All patients underwent diagnostic laparoscopy with peritoneal lavage and biopsies. Patients were stratified according to the Peritoneal Cancer Index (PCI) and directed to appropriate treatment pathways, including CRS + HIPEC, PIPAC combined with systemic chemotherapy, or neoadjuvant chemotherapy followed by surgical re-evaluation. **Results**: Occult peritoneal metastases were detected in 20% of patients (80/400). Among patients with peritoneal metastasis, 70 patients ultimately underwent CRS + HIPEC and 39 underwent PIPAC-based treatment. Among these 70 patients, 53 patients with PCI < 7 were initially allocated to CRS + HIPEC and an additional 17 patients with newly detected limited peritoneal disease (PCI < 7) underwent CRS + HIPEC following restaging laparoscopy after neoadjuvant chemotherapy, resulting in a total of 70 patients in the CRS + HIPEC group. In the CRS + HIPEC group, the 1-year survival rate was 70% with a morbidity rate of 25.7%. In the PIPAC group, the 1-year survival rate was 50% with a morbidity rate of 25.6%. Among patients receiving neoadjuvant chemotherapy, disease progression was detected in 14% during restaging laparoscopy. Palliative surgery provided effective symptom control in inoperable patients, with a 1-year survival rate of approximately 15%. **Conclusions**: Diagnostic laparoscopy provides additional staging information and guides treatment allocation in patients with advanced gastric cancer. PCI-based assessment enables individualized selection of CRS + HIPEC, PIPAC, or systemic treatment strategies. However, the present study was not designed to determine whether DL-guided treatment allocation improves survival, and the observed survival outcomes should therefore be interpreted as descriptive real-world outcomes rather than evidence of treatment benefit.

## 1. Introduction

Gastric cancer (GC) is a malignancy with high incidence and mortality worldwide, particularly in East Asia and Eastern Europe [1,2]. The prognosis of the disease largely depends on the stage at diagnosis, with significantly improved survival rates in patients diagnosed at an early stage [3]. However, most patients are diagnosed at an advanced stage, and peritoneal dissemination is frequently observed [4,5].

Peritoneal carcinomatosis (PC) is one of the most common routes of metastatic spread in GC and has a substantial impact on patients’ eligibility for surgical treatment [6,7]. Imaging modalities, particularly computed tomography (CT) and magnetic resonance imaging (MRI), are effective in detecting large lesions but often fail to identify low-volume peritoneal metastases [8,9]. Therefore, diagnostic laparoscopy (DL) is considered the gold standard for detecting occult peritoneal disease [10,11].

In recent years, advanced treatment modalities such as cytoreductive surgery (CRS), hyperthermic intraperitoneal chemotherapy (HIPEC), and pressurized intraperitoneal aerosol chemotherapy (PIPAC) have enabled more accurate patient selection and optimization of treatment strategies [12,13,14,15,16]. The aim of this study is to retrospectively evaluate the impact of DL on clinical decision-making in GC staging in a large cohort of 400 patients, as well as to analyze treatment distribution according to the peritoneal carcinomatosis index (PCI) score and post-treatment outcomes [17,18].

## 2. Methods

### 2.1. Study Design and Ethical Approval

This study was designed as a retrospective cohort analysis. Ethical approval was obtained from the Institutional Ethics Committee (approval number: 13.06.2024/186). All procedures were conducted in accordance with the ethical standards of the institutional and/or national research committee and with the 1964 Declaration of Helsinki and its later amendments. All patients were evaluated by a multidisciplinary tumor board during the preoperative period.

### 2.2. Patient Selection

A total of 400 patients diagnosed with gastric adenocarcinoma between June 2016 and March 2026, who were deemed suitable for surgical intervention or laparoscopic staging, were included in the study. To ensure a valid 1-year survival analysis, only patients who were enrolled at least 12 months prior to the final data cutoff (i.e., treated between June 2016 and March 2025) were included in the 1-year overall survival calculations, whereas patients included after March 2025 were included solely for clinical and perioperative safety analyses. The exclusion criteria were as follows: History of a previously diagnosed or treated malignancy, initiation of systemic therapy prior to surgical evaluation and patients deemed unfit for laparoscopy due to severe cardiopulmonary comorbidities.

### 2.3. Data Collection and Variables

Demographic data (age, sex, and ECOG performance status), tumor location, histopathological type, PCI score, laparoscopic findings, and administered treatment modalities were retrospectively collected from patient records. The PCI score was calculated according to Sugarbaker’s criteria. PCI was assessed intraoperatively by systematic inspection of the 13 abdominopelvic regions defined in the Sugarbaker classification. Each region was assigned a lesion size score from 0 to 3, and the total PCI score was calculated as the sum of the regional scores, ranging from 0 to 39. For treatment allocation, a low PCI was defined as <7 and a high PCI as ≥7. Patients with a low PCI (<7) were considered candidates for CRS + HIPEC, whereas those with a high PCI (≥7) were allocated to PIPAC combined with systemic chemotherapy. All laparoscopic procedures were performed by experienced surgical oncology surgeons.

### 2.4. Laparoscopic Procedure and Treatment Allocation

All patients underwent a standard three-port diagnostic laparoscopy under general anesthesia using a high-definition laparoscopic video system (Sony Corporation, Tokyo, Japan). Pneumoperitoneum was established with carbon dioxide at an intra-abdominal pressure of 12 mmHg. A camera port was inserted through the umbilical or periumbilical region, followed by placement of two working ports in the right and left lower quadrants according to the patient’s body habitus and the anticipated extent of exploration.

A systematic inspection of all 13 abdominopelvic regions was performed according to the Sugarbaker Peritoneal Cancer Index (PCI) classification. The liver surface, diaphragmatic peritoneum, peritoneal gutters, pelvis, omentum, stomach, small bowel, mesentery, and all accessible peritoneal surfaces were carefully evaluated for metastatic implants. Before manipulation of the abdominal cavity, peritoneal lavage was performed using sterile saline, and lavage fluid was collected for cytological examination. Four-quadrant peritoneal biopsies and additional biopsies from suspicious lesions were obtained routinely. Cytological specimens were examined by experienced cytopathologists, whereas tissue specimens underwent standard histopathological evaluation.

Patients were managed according to a predefined institutional treatment algorithm based on the presence of peritoneal metastasis and the PCI score. Patients without evidence of peritoneal metastasis (PCI = 0) received standard neoadjuvant chemotherapy followed by reassessment for curative gastrectomy. Patients with histologically or cytologically confirmed peritoneal metastasis and a PCI score < 7 were considered candidates for cytoreductive surgery (CRS) combined with hyperthermic intraperitoneal chemotherapy (HIPEC). Patients with a PCI score ≥ 7 were treated with pressurized intraperitoneal aerosol chemotherapy (PIPAC) in combination with systemic chemotherapy. Patients with diffuse small bowel involvement, extensive distant metastases, or poor performance status precluding oncological treatment received palliative treatment according to multidisciplinary tumor board recommendations.

The PCI threshold of 7 was selected a priori according to international consensus recommendations, including the PSOGI/Chicago Consensus and the International Delphi Consensus for gastric cancer with synchronous peritoneal metastases, which identify PCI ≤ 7 as an appropriate threshold for considering complete cytoreduction in carefully selected patients.

All laparoscopic procedures were performed by board-certified surgical oncologists who had completed general surgery residency followed by a two-year subspecialty fellowship in Surgical Oncology. The study was conducted in a tertiary referral Surgical Oncology Center performing approximately 400 oncological operations annually, including more than 400 CRS + HIPEC procedures since 2016.

### 2.5. Chemotherapy Protocols According to PCI Score

Patients were stratified by tumor stage and intraoperative PCI score to guide neoadjuvant and palliative chemotherapy. Patients without peritoneal involvement received standard neoadjuvant systemic therapy, a FLOT regimen (5-fluorouracil, oxaliplatin, docetaxel, calcium folinate) with a median of 4–6 cycles. Patients were scheduled for surgery approximately 4–6 weeks after completion of neoadjuvant therapy. Adjuvant chemotherapy continued postoperatively for a median of 4–6 cycles in patients undergoing curative resection [19,20,21,22]. Adjuvant chemotherapy was provided only for patients achieving resectability, while unresectable or progressive cases continued systemic therapy as tolerated [23,24,25,26,27,28,29].

Patients with synchronous peritoneal metastasis and a PCI score < 7 received systemic chemotherapy followed by cytoreductive surgery (CRS) combined with hyperthermic intraperitoneal chemotherapy (HIPEC) when complete or near-complete cytoreduction (CC-0/CC-1) was considered technically feasible. HIPEC was performed immediately after CRS using the closed-abdomen technique. Hyperthermic intraperitoneal chemotherapy was administered at 42 °C for 60 min using either Mitomycin-C or Oxaliplatin according to institutional treatment protocols, tumor characteristics, and multidisciplinary tumor board recommendations.

Patients with a PCI score ≥ 7 underwent pressurized intraperitoneal aerosol chemotherapy (PIPAC) combined with systemic chemotherapy. PIPAC was performed laparoscopically using cisplatin (10.5 mg/m^2^) and doxorubicin (2.1 mg/m^2^), delivered as aerosolized chemotherapy through a CE-certified nebulizer (CapnoPen^®^, Capnomed GmbH, Villingendorf, Germany) under a capnoperitoneum of 12 mmHg at 37 °C for 30 min. A minimum of three PIPAC cycles was planned for each patient, with repeat procedures performed at 6- to 8-week intervals according to radiological evaluation, laparoscopic reassessment, pathological response assessment, treatment tolerance, and patient performance status. Additional PIPAC cycles were considered in patients demonstrating clinical benefit, disease control, or objective response without unacceptable toxicity.

Systemic treatment selection was individualized according to tumor characteristics, treatment history, performance status, organ function, and multidisciplinary tumor board recommendations. Systemic chemotherapy regimens for metastatic disease included FOLFOX (5-fluorouracil, leucovorin, and oxaliplatin), CAPEOX (capecitabine and oxaliplatin), or FOLFIRI (5-fluorouracil, leucovorin, and irinotecan), selected according to previous treatment exposure, organ function, treatment tolerance, and multidisciplinary tumor board recommendations.

Systemic treatment selection was individualized according to tumor characteristics, molecular profile, performance status, and multidisciplinary tumor board recommendations. Targeted therapies or immune checkpoint inhibitors were administered when clinically indicated according to guideline recommendations.

### 2.6. Assessment of Perioperative Morbidity and Mortality

Postoperative complications were graded according to the Clavien–Dindo classification. Perioperative morbidity was defined as any complication occurring during the postoperative period and before hospital discharge or during the predefined early postoperative follow-up period. Major morbidity was defined as a Clavien–Dindo grade III or higher complication. Perioperative mortality was defined as death occurring within 30 days after surgery or during the index hospital admission, whichever occurred later.

### 2.7. Follow-Up

Patients were followed every three months during the first two postoperative years, every six months during years 3–5, and annually thereafter. Follow-up evaluations included physical examination, laboratory investigations, thoracoabdominal computed tomography, and upper gastrointestinal endoscopy when clinically indicated. Follow-up data were obtained from routine outpatient visits, institutional electronic medical records, and telephone interviews. The median follow-up duration for the entire cohort was 45 months (range, 5–109 months).

Overall survival (OS) was defined as the interval from the date of the initial diagnostic laparoscopy, which represented the baseline treatment allocation, to death from any cause or the last follow-up visit. Disease-free survival (DFS) was evaluated only in patients who underwent curative-intent gastrectomy and was defined as the interval from curative surgery to the first documented recurrence or death from any cause. Patients who were alive without an event at the last follow-up were censored at the date of their last clinical assessment.

### 2.8. Handling of Missing Data

Data completeness was assessed for all study variables before statistical analysis. When available, missing information was obtained from electronic medical records and institutional databases. Variables with unavailable data were analyzed based on the number of patients with available observations, and no values were imputed. The number of patients included in each analysis was therefore reported according to data availability where applicable.

### 2.9. Statistical Analysis

Statistical analyses were performed using IBM SPSS Statistics version 25.0 (IBM Corp., Armonk, NY, USA). Continuous variables are presented as mean ± standard deviation (SD) or median (interquartile range [IQR]) according to data distribution, whereas categorical variables are presented as frequencies and percentages. Comparisons between categorical variables were performed using the Chi-square test or Fisher’s exact test, as appropriate. Continuous variables were compared using the independent-samples *t*-test or Mann–Whitney U test depending on data distribution.

Overall survival (OS) and disease-free survival (DFS) were estimated using the Kaplan–Meier method and compared with the log-rank test. Only patients with at least 12 months of potential follow-up (treated before March 2025) were included in the predefined one-year survival analyses. The Cox proportional hazards analyses were performed using the entire study cohort with time-to-event censoring at the last available follow-up. The 12-month eligibility criterion applied only to the calculation of the predefined one-year survival rates and did not affect the Cox regression analyses.

Variables with a *p*-value < 0.10 in univariable Cox regression analysis together with predefined clinically relevant variables (age, sex, ECOG performance status, histological subtype, tumor location, and baseline PCI) were entered into a multivariable Cox proportional hazards regression model. TNM stage was not included in the multivariable analysis because of collinearity with the T and N stage variables. Hazard ratios (HRs) with 95% confidence intervals (CIs) were calculated.

The proportional hazards assumption was assessed graphically using log-minus-log survival plots and was not violated for the final Cox regression model. All statistical tests were two-sided, and *p*-values < 0.05 were considered statistically significant. Given the retrospective observational design and non-randomized treatment allocation, survival comparisons between treatment groups were considered exploratory and descriptive. Treatment selection was based on baseline disease characteristics, particularly peritoneal disease burden and PCI score; therefore, these comparisons were not interpreted as direct estimates of treatment efficacy.

## 3. Results

### 3.1. Patient Flow and Selection

**Figure 1:**
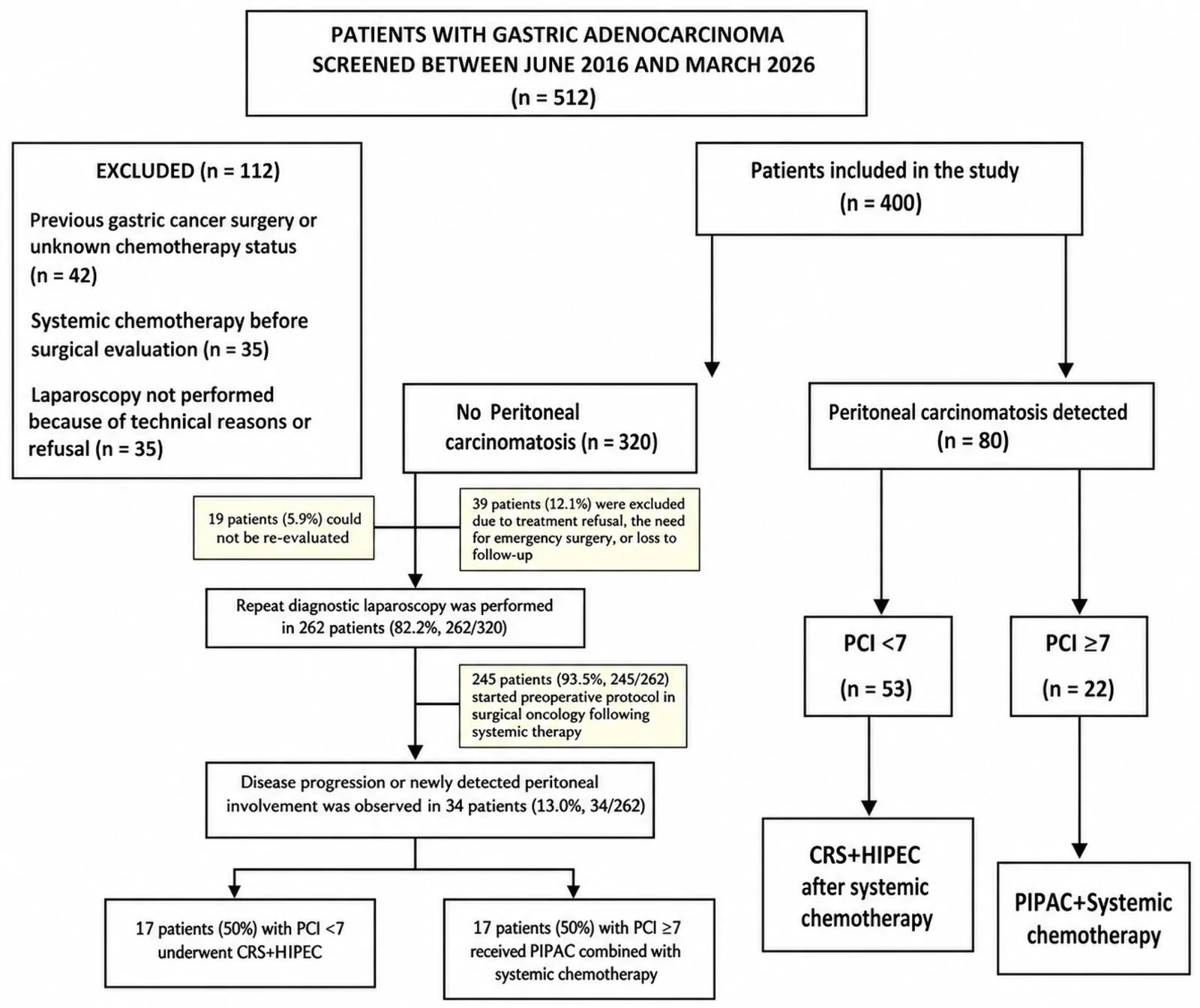
Patient flowchart detailing exclusions, diagnostic laparoscopy findings, repeat laparoscopy, and subsequent treatment allocation.

A total of 512 patients with gastric cancer (GC) were screened from the institutional clinical database between June 2016 and March 2026. Patients with a history of previous gastric cancer surgery or unknown chemotherapy status (*n* = 42), those who had received systemic chemotherapy before surgical evaluation (*n* = 35), and patients in whom diagnostic laparoscopy could not be performed because of technical limitations or refusal (*n* = 35) were excluded. Consequently, 112 patients were excluded, and 400 consecutive patients constituted the final study cohort. The patient selection process, diagnostic laparoscopy findings, and subsequent treatment allocation are summarized in Figure 1.

### 3.2. Demographic and Clinical Characteristics

The baseline characteristics of the study population are summarized in Table 1. The mean age was 61.4 ± 11.2 years, and 262 patients (65.5%) were male. Most patients (72%) had an ECOG performance status of 0–1. Histopathological examination demonstrated diffuse-type adenocarcinoma in 192 patients (48%) and intestinal-type adenocarcinoma in 208 patients (52%). Tumors were most commonly located in the antrum (60%), followed by the corpus (25%), fundus (10%), and gastroesophageal junction (5%). The mean number of examined lymph nodes during diagnostic staging was 6.3 ± 2.1.

### 3.3. Initial Laparoscopy and PCI Distribution

All 400 patients underwent diagnostic laparoscopy, peritoneal washing cytology, and systematic four-quadrant peritoneal biopsies. Previously occult peritoneal metastases were identified in 80 patients (20%), all of whom had histological and/or cytological confirmation of peritoneal dissemination. Among patients with peritoneal metastasis, the mean PCI score was 6.2 ± 3.1 (range, 1–18).

Of these 80 patients, 53 (66.3%) had PCI < 7 and were considered candidates for CRS plus HIPEC according to the institutional treatment algorithm. Twenty-two patients (27.5%) presented with PCI ≥ 7 and received PIPAC combined with systemic chemotherapy. During restaging laparoscopy following neoadjuvant chemotherapy, an additional 17 patients were found to have newly developed peritoneal metastases with PCI ≥ 7 and were subsequently allocated to PIPAC combined with systemic chemotherapy, resulting in a final PIPAC cohort of 39 patients (22 initial and 17 restaged patients) Five patients (6.3%) had extensive disease burden or poor performance status and therefore received best supportive care without disease-directed oncological treatment.

The remaining 320 patients showed no evidence of peritoneal metastasis (PCI = 0) and were scheduled for neoadjuvant chemotherapy before reassessment for curative surgery (Table 2).

Patients initially considered candidates for CRS + HIPEC after diagnostic laparoscopy included 53 patients; an additional 17 patients underwent CRS + HIPEC after restaging laparoscopy following neoadjuvant chemotherapy.

### 3.4. Neoadjuvant Therapy, Repeat Laparoscopy, and Disease Progression

Among the 320 patients initially allocated to neoadjuvant chemotherapy, 261 (81.6%) completed the planned treatment protocol, whereas 59 patients (18.4%) did not complete neoadjuvant therapy because of treatment refusal, emergency surgery, deterioration in performance status, or loss to follow-up (Table 3).

Of the 261 patients who completed neoadjuvant chemotherapy, 242 underwent repeat staging laparoscopy. Disease progression or newly developed peritoneal metastasis was detected in 34 patients (14.0%), resulting in treatment modification. Seventeen patients with PCI < 7 underwent CRS plus HIPEC, whereas 17 patients with PCI ≥ 7 received PIPAC combined with systemic chemotherapy. The remaining 208 patients proceeded to curative gastrectomy.

Patients who did not complete neoadjuvant chemotherapy or who became unsuitable for curative treatment because of progressive disease subsequently underwent individualized management consisting of palliative systemic therapy, best supportive care, or palliative surgical procedures according to multidisciplinary tumor board recommendations. Overall, 39 patients ultimately required palliative surgical intervention because of gastric outlet obstruction, bleeding, intestinal obstruction, or inability to maintain adequate oral intake.

### 3.5. Neoadjuvant Therapy-Related Complications

Treatment-related adverse events are summarized in Table 4. Grade 3–4 hematological toxicity occurred in 39 patients (15%), gastrointestinal intolerance in 33 (12.6%), partial bowel obstruction in 7 (2.7%), and minor gastrointestinal bleeding in 5 (1.9%). All toxicities were managed conservatively with dose modifications or temporary treatment interruption, and no treatment-related mortality occurred.

### 3.6. Surgical Outcomes

Among the patients initially classified without peritoneal metastasis (PCI = 0), 242 patients underwent restaging laparoscopy after completion of neoadjuvant chemotherapy. During restaging evaluation, newly developed limited peritoneal metastases were detected in 34 patients. Seventeen of these patients had a PCI score < 7 and subsequently underwent CRS plus HIPEC. Therefore, the final CRS plus HIPEC cohort consisted of 70 patients, including 53 patients diagnosed with synchronous peritoneal metastasis at initial diagnostic laparoscopy and 17 patients identified during restaging laparoscopy after neoadjuvant chemotherapy. Among the 70 patients who underwent CRS plus HIPEC, the mean PCI was 5.3 ± 1.1. The overall postoperative morbidity rate was 25.7%, including anastomotic leakage (5.7%), postoperative infection (10.0%), bowel obstruction (4.3%), and hematological toxicity (5.7%). Two patients died during the postoperative period, corresponding to a surgical mortality rate of 2.9% (Table 5).

Thirty-nine patients underwent PIPAC. The mean PCI in this group was 10.2 ± 2.0. The overall postoperative morbidity rate was 25.6%, with nausea/vomiting (15.4%), transient hematological toxicity (7.7%), and minor intra-abdominal bleeding (2.6%) being the most frequent complications. The final PIPAC cohort consisted of 39 patients, including 22 patients diagnosed with peritoneal metastasis at initial laparoscopy and 17 patients identified during restaging laparoscopy (Table 6).

Overall, 39 patients underwent palliative surgical procedures because of progressive disease resulting in gastric outlet obstruction, intestinal obstruction, tumor-related bleeding, or inability to tolerate oral intake. These patients originated from both the group that failed to complete neoadjuvant treatment and those who subsequently developed unresectable disease during follow-up. Overall postoperative morbidity occurred in 9 patients (23.1%), while no procedure-related mortality attributable to palliative surgery was observed (Table 7).

### 3.7. Intraoperative Findings and Procedure Safety

Minor intraoperative complications occurred in 6 patients (1.5%), including small-vessel injury (*n* = 3), liver capsule contusion (*n* = 2), and splenic capsule injury (*n* = 1). All complications were managed laparoscopically without conversion to open surgery. No visceral perforation, major hemorrhage requiring transfusion, port-site complications, reoperation, or procedure-related mortality occurred. The mean operative time for diagnostic laparoscopy was 38 ± 12 min.

### 3.8. Survival Outcomes

**Figure 2:**
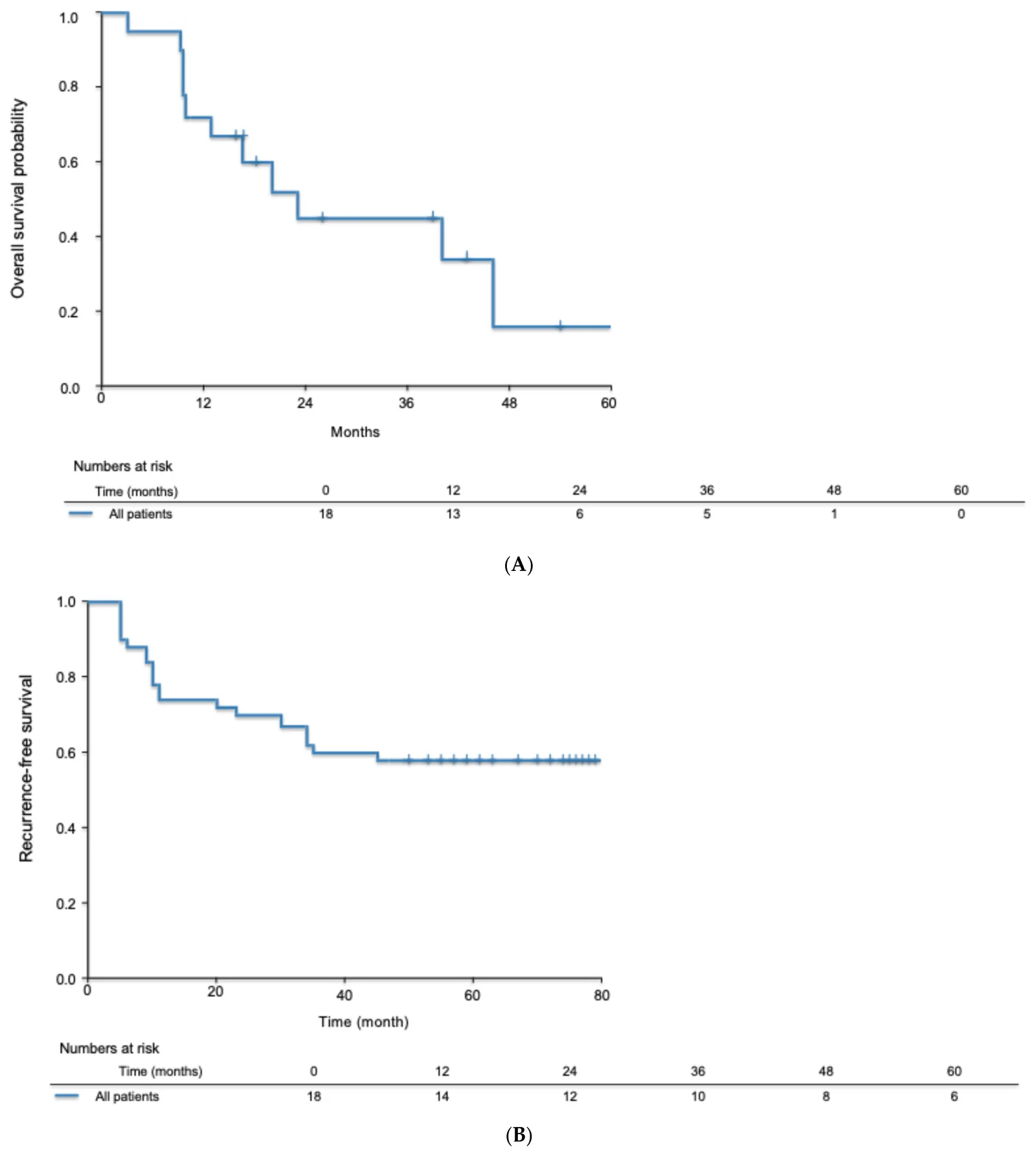
(**A**) Kaplan–Meier Overall Survival Curve. (**B**) Kaplan–Meier Disease-Free Survival Curve.

As a secondary analysis, overall survival was evaluated descriptively to characterize real-world outcomes according to the treatment strategies determined by diagnostic laparoscopy and PCI-based treatment allocation. The median follow-up duration was 45 months (IQR, 28–67 months). The median overall survival (OS) for the entire study cohort was 56 months, whereas the median disease-free survival (DFS) was 40 months. The estimated 1-, 3-, and 5-year OS rates for the entire cohort were 85%, 65%, and 46%, respectively, while the corresponding DFS rates were 70%, 50%, and 36% (Figure 2A,B).

To illustrate survival according to treatment strategy, Kaplan–Meier survival curves were generated for the three principal treatment groups: CRS plus HIPEC (*n* = 70), PIPAC combined with systemic chemotherapy (*n* = 39), and palliative surgery (*n* = 39). The corresponding numbers at risk are presented beneath the survival curves, and survival distributions were compared using the log-rank test.

**Figure 3:**
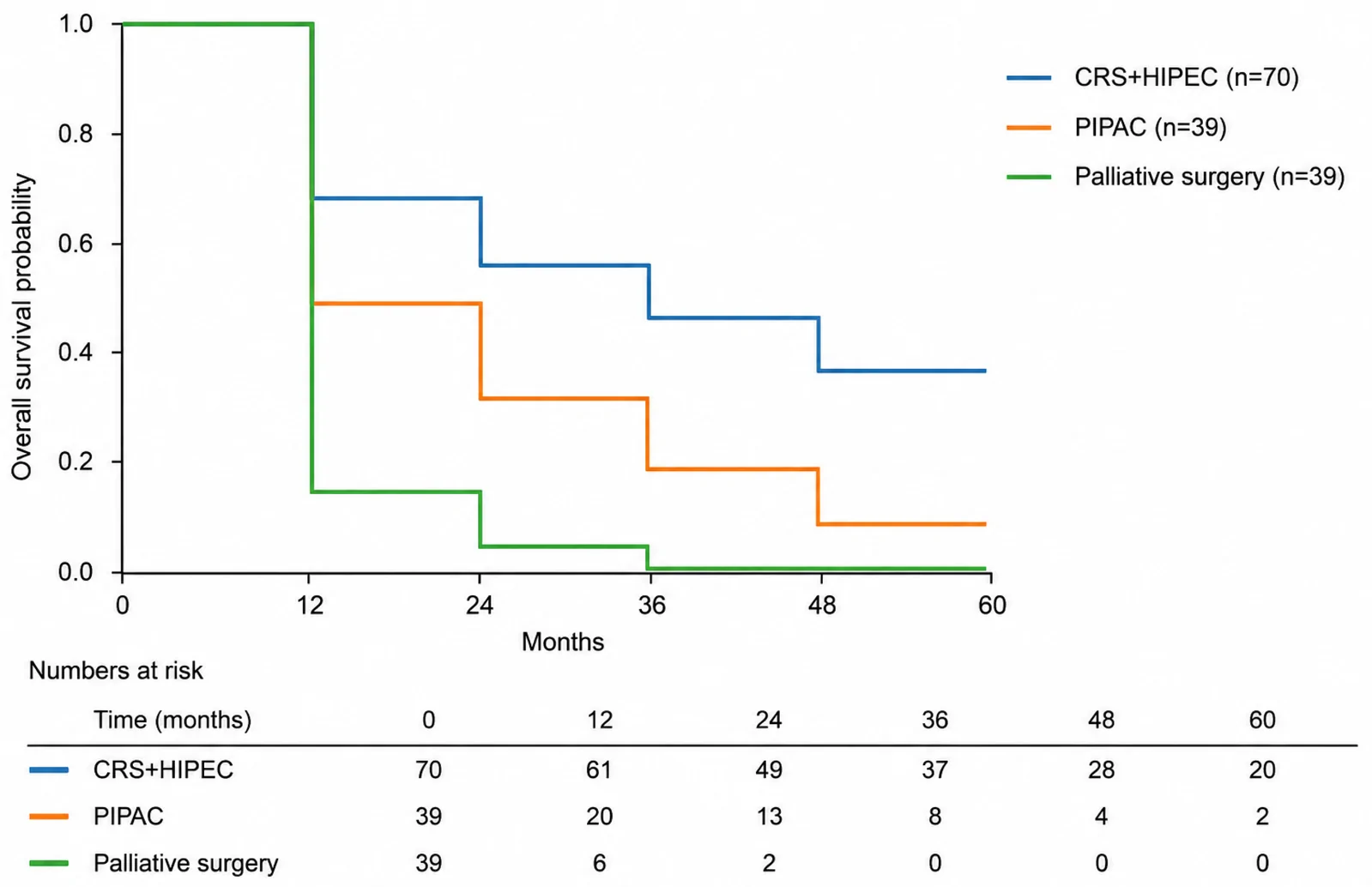
Overall Survival According to Treatment Strategy.

Patients treated with CRS plus HIPEC demonstrated the most favorable survival, whereas those receiving PIPAC showed intermediate outcomes. Palliative surgery was associated with the poorest survival, reflecting the advanced stage of disease and limited therapeutic options in this patient population. The median OS was not reached in the CRS plus HIPEC group, was approximately 12 months in the PIPAC group, and was less than 12 months in the palliative surgery group. The corresponding one-year OS rates were 70%, 50%, and 15%, respectively (Table 5, Table 6 and Table 7). Kaplan–Meier analysis demonstrated significant differences in overall survival among the three treatment groups (log-rank *p* < 0.001) (Figure 3).

**Figure 4:**
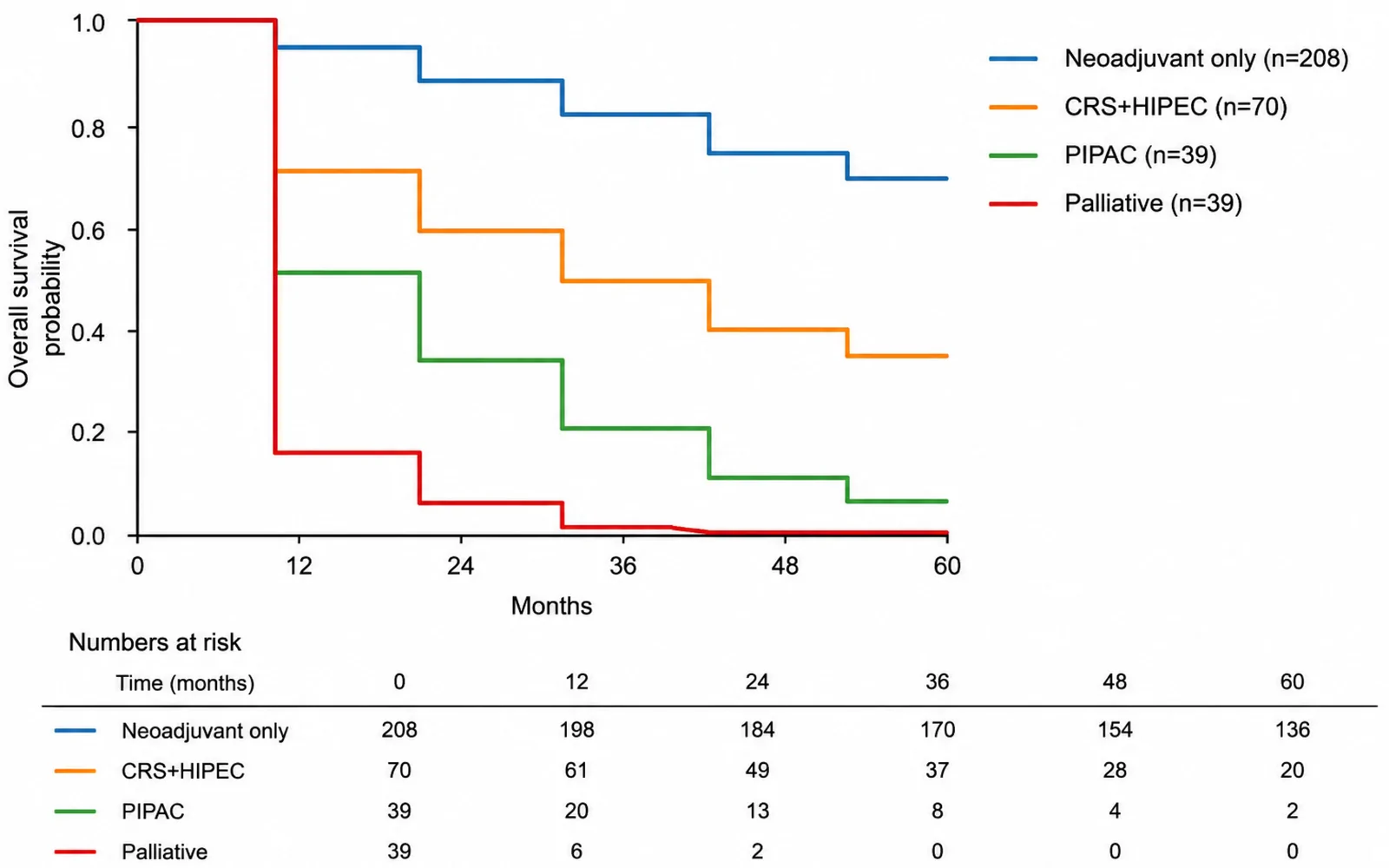
Overall Survival Including the Reference Neoadjuvant Group.

As recommended by the reviewer, patients without peritoneal metastasis who completed neoadjuvant chemotherapy followed by curative gastrectomy were additionally included as a reference cohort (Figure 4). This group demonstrated the most favorable long-term survival compared with the treatment groups. Because treatment allocation was determined according to baseline disease burden, particularly the presence of peritoneal metastasis and the baseline Peritoneal Cancer Index (PCI), these survival comparisons should be interpreted as descriptive real-world outcomes rather than evidence of comparative treatment efficacy.

### 3.9. Prognostic Factors

Univariable Cox regression analysis was performed for all predefined clinicopathological variables. Tumor size, ECOG performance status, baseline PCI, T stage, N stage, TNM stage, histological grade, and lymphovascular invasion were significantly associated with overall survival (Table 8). Age, sex, histological subtype, and tumor location were not significantly associated with survival in the univariable analysis.

Variables with a *p*-value < 0.10 in the univariable analysis together with predefined clinically relevant covariates (age, sex, ECOG performance status, histological subtype, tumor location, and baseline PCI) were entered into a multivariable Cox proportional hazards regression model. TNM stage was excluded because of collinearity with T and N stage variables.

In the full multivariable Cox regression model, ECOG performance status, baseline PCI, T stage, and N stage remained independently associated with overall survival. Other variables, including age, sex, histological subtype, tumor location, tumor size, histological grade, and lymphovascular invasion, did not retain statistical significance after adjustment (Table 9).

The proportional hazards assumption was satisfied for the final model. After multivariable adjustment, ECOG performance status, baseline PCI, T stage, and N stage remained independent predictors of overall survival. Although tumor size, histological grade, and lymphovascular invasion were significant in univariable analysis, their associations were attenuated after adjustment and did not remain statistically significant in the full Cox regression model.

## 4. Discussion

Diagnostic laparoscopy plays a pivotal role in the staging and management of GC, particularly in detecting occult peritoneal metastases that cannot be identified by conventional imaging modalities such as CT or MRI. Owing to its high sensitivity in identifying low-volume peritoneal disease, DL is widely considered the gold standard in staging advanced GC [30,31]. Previous studies have reported detection rates of occult peritoneal metastasis ranging between 15% and 25% [32,33]. In our cohort, the detection rate was 20%, which is consistent with the literature and reinforces the clinical value of DL. The relatively large sample size of our study further strengthens the reliability of these findings and supports the integration of DL into routine staging algorithms. Importantly, the clinical relevance of DL in our cohort was reflected not only by the detection of occult peritoneal disease but also by its direct effect on disease classification and treatment allocation. All patients were considered to have potentially resectable, non-metastatic advanced gastric cancer based on preoperative clinical and radiological assessment before DL. However, DL identified occult peritoneal disease in 80 patients (20%), and peritoneal involvement was confirmed by peritoneal biopsy and/or positive cytology in all of these patients. These patients were consequently reclassified as having stage IV disease and were redirected from a potentially curative treatment pathway to systemic treatment strategies appropriate for metastatic disease. Therefore, although the absence of a comparator group prevents us from determining the causal effect of DL on survival or other clinical outcomes, these findings demonstrate that DL provided additional staging information that changed disease classification and treatment allocation in a substantial proportion of patients. The survival analysis further emphasized the prognostic relevance of the peritoneal findings identified by DL. Patients without peritoneal metastasis who completed neoadjuvant chemotherapy followed by curative gastrectomy demonstrated the most favorable long-term survival trajectory compared with patients requiring CRS plus HIPEC, PIPAC, or palliative surgery (Figure 4). Although these comparisons are descriptive and should not be interpreted as evidence of treatment efficacy, the marked differences in survival according to the presence and extent of peritoneal disease reinforce the prognostic importance of DL findings and PCI-based disease assessment.

For patients with a PCI score < 7, CRS combined with HIPEC has been shown to improve locoregional control and survival outcomes [34,35]. Glehen et al. and Yonemura et al. reported 3-year survival rates of approximately 50–60% and morbidity rates between 20% and 30% in selected patients undergoing CRS + HIPEC [34,35]. In our study, the CRS + HIPEC group demonstrated a 1-year survival rate of 70% and a postoperative morbidity rate of 25.7%, which are in line with previously reported outcomes. These findings highlight the importance of appropriate patient selection based on PCI. However, because patients undergoing CRS plus HIPEC had a lower baseline disease burden than those treated with PIPAC, the observed survival outcomes should be interpreted as descriptive real-world clinical outcomes rather than evidence of treatment superiority. Furthermore, continuation of systemic chemotherapy regimens such as FLOT, CAPOX, or FOLFOX in the perioperative setting may have contributed to improved outcomes in this group [19,20,21,22].

In patients with PCI ≥ 7, combined treatment with PIPAC and systemic chemotherapy offers a less invasive yet effective alternative for disease control. Previous studies have reported 1-year survival rates of approximately 50–60% and relatively low morbidity rates in patients treated with PIPAC [3,36]. Consistent with these findings, our cohort demonstrated a 1-year survival rate of 50% and an overall morbidity rate of 25.6%. Although the morbidity observed in our study appears slightly higher, this may be attributed to patient selection, disease burden, and real-world clinical conditions. These findings support the feasibility and safety of PIPAC as a therapeutic option in advanced-stage gastric cancer within appropriately selected patients according to disease burden and PCI score.

Palliative surgical interventions remain essential in patients deemed inoperable following neoadjuvant therapy, particularly for symptom control such as obstruction or bleeding. Previous studies have emphasized the role of palliative surgery in maintaining quality of life despite limited survival outcomes [4]. Additionally, restaging laparoscopy following neoadjuvant chemotherapy has been shown to reduce unnecessary laparotomies and improve treatment decision-making [37,38]. In our study, 39 patients underwent palliative surgery, achieving effective symptom control and a 1-year survival rate of approximately 15%, consistent with the aggressive nature of advanced gastric cancer with peritoneal metastases. These findings further highlight the clinical importance of DL in guiding appropriate treatment pathways.

Surgeon experience is a critical determinant of the diagnostic accuracy of DL. It has been reported that experienced surgical teams can increase the detection rate of occult peritoneal metastases by up to 10–15% [37,38]. In our study, all laparoscopic procedures were performed by experienced surgeons, and peritoneal disease was detected in 20% of patients, supporting the reliability of our findings and emphasizing the importance of surgical expertise in staging procedures. Although advances in minimally invasive and robotic surgical platforms have demonstrated favourable safety and efficacy profiles in gastric cancer surgery, the choice of surgical platform does not eliminate the need for accurate peritoneal staging. DL remains an essential component of the diagnostic work-up for appropriately selected patients, irrespective of whether subsequent definitive surgery is performed using an open, laparoscopic, or robotic approach. Robotic gastrectomy may nevertheless influence future treatment algorithms by improving surgical dexterity, three-dimensional visualization, and the precision of lymphadenectomy and complex intracorporeal reconstruction. A recent systematic review and meta-analysis by Li et al. demonstrated that robotic gastrectomy was associated with favourable perioperative outcomes compared with laparoscopic gastrectomy, including reduced blood loss, lower postoperative complication rates, increased lymph-node retrieval, and faster postoperative recovery, although operative times were longer [39]. These advances may facilitate the broader adoption of minimally invasive approaches in technically complex gastric cancer surgery and may allow more individualized treatment pathways in the future. However, the potential advantages of robotic platforms should be considered complementary to, rather than a replacement for, systematic peritoneal staging. Future treatment algorithms may therefore integrate diagnostic laparoscopy and PCI-based assessment with the selection of the most appropriate definitive surgical platform, including robotic surgery, according to disease extent, anatomical complexity, institutional expertise, and available resources. Prospective comparative studies are needed to determine whether robotic platforms can meaningfully influence staging-to-treatment pathways and long-term oncological outcomes in patients with advanced gastric cancer.

From a health economics perspective, DL provides significant cost-effectiveness by preventing unnecessary laparotomies and enabling appropriate patient selection for multimodal treatment strategies [40,41,42]. Previous studies have demonstrated that early detection of peritoneal metastases reduces both clinical burden and healthcare costs [41,42]. Similarly, in our cohort, DL facilitated accurate treatment allocation and avoided non-beneficial surgical interventions, contributing to both clinical and economic benefits.

Although diagnostic laparoscopy remains the most definitive method for direct assessment of the peritoneal cavity, emerging artificial intelligence (AI)-based technologies may provide valuable complementary support across the diagnostic pathway of gastrointestinal malignancies. AI-assisted imaging systems have demonstrated potential in improving the detection and characterization of gastrointestinal lesions on cross-sectional imaging and endoscopy, while AI-based endoscopic tools may enhance the recognition of subtle mucosal abnormalities and support more standardized diagnostic interpretation. In addition, AI-driven decision-support systems may facilitate the integration of clinical, radiological, endoscopic, and pathological data to improve risk stratification and treatment planning in patients with gastrointestinal cancers. Recent reviews have emphasized the expanding role of AI in gastrointestinal disease, particularly in image interpretation, endoscopic diagnosis, lesion detection, and clinical decision support, highlighting its potential to improve diagnostic accuracy and enable more individualized clinical decision-making [43,44].

Nevertheless, the cost-effectiveness of routine DL should be considered within the context of local healthcare resources. Although DL may reduce the costs associated with unnecessary laparotomy and inappropriate treatment allocation, its routine implementation also requires additional operating-room time, anaesthesia, laparoscopic equipment, perioperative care, and appropriately trained surgical personnel. These costs may represent a substantial barrier in resource-limited centres, particularly where access to advanced laparoscopic infrastructure or experienced surgeons is limited. Therefore, the clinical and economic value of DL may vary according to institutional resources, disease prevalence, local treatment pathways, and healthcare reimbursement systems. In such settings, a selective approach based on clinical stage, radiological findings, tumour characteristics, and the anticipated likelihood of occult peritoneal disease may be more feasible than universal routine implementation. Accordingly, the integration of DL into staging algorithms should ideally be accompanied by formal cost-effectiveness analyses that consider both the direct costs of the procedure and the potential savings resulting from the avoidance of unnecessary laparotomy and non-beneficial treatment.

The strengths of this study include a relatively large sample size (*n* = 400), systematic application of DL in all patients, and standardized PCI assessment performed by experienced surgeons. However, several limitations should be acknowledged. The retrospective and single-center design may limit the generalizability of the findings. Since this study was primarily designed to emphasize the role of diagnostic laparoscopy in guiding the treatment pathway in gastric cancer, detailed data on the toxicities of the administered individual chemotherapies were not included. However all patients received guideline-concordant standard systemic treatments and no treatment discontinuations due to toxicity were observed in any patient. Additionally, the relatively short follow-up period in some patients restricts long-term survival analysis. Variability in surgical expertise and treatment strategies across different centers may also influence outcomes, and subjective clinical decision-making in multimodal treatment planning cannot be entirely excluded.

The survival outcomes reported in the present study should be interpreted within the context of the study design. Patients undergoing CRS plus HIPEC and those receiving PIPAC represented clinically distinct populations with different baseline disease burdens, particularly regarding PCI score and resectability. Therefore, the observed survival outcomes reflect descriptive real-world clinical experience rather than comparative treatment efficacy. Prospective multicenter studies incorporating multivariable adjustment or propensity score-based analyses are warranted to determine the comparative effectiveness of these treatment strategies.

Future prospective, multicenter studies are warranted to better define the impact of DL and PCI-based treatment strategies on long-term survival, morbidity, and quality of life [32,33]. Further research should focus on optimizing patient selection criteria, refining PIPAC and CRS + HIPEC protocols, and evaluating the role of combined and personalized therapeutic approaches. Moreover, cost-effectiveness analyses and the integration of novel systemic therapies may further enhance treatment outcomes in advanced GC [3,36].

## 5. Conclusions

DL is an indispensable method that enhances staging accuracy in GC and plays a critical role in guiding treatment strategies. In our study, the detection of occult peritoneal metastases in 20% of patients clearly highlights the importance of diagnostic laparoscopy in the clinical decision-making process. Early identification of peritoneal disease may help reduce unnecessary laparotomies and enables patients to be directed toward multimodal treatment strategies according to disease burden. PCI-based patient selection for CRS + HIPEC and PIPAC was associated with distinct real-world survival outcomes and acceptable morbidity rates in selected patient groups. However, given the retrospective and non-randomized study design, the survival benefit of DL-guided treatment allocation cannot be directly determined from our data. Accordingly, DL should be considered an integral component of staging algorithms in appropriately selected patients with advanced GC.

## Figures and Tables

**Table 1:** Baseline Demographic and Clinical Characteristics of the Study Population.

Parameter	*n*	%/Mean ± SD
Age (years)	–	61.4 ± 11.2
Sex		
–Male	262	65.5
–Female	138	34.5
ECOG Performance		
–0–1	288	72
–2–3	112	28
Histopathology		
–Diffuse	192	48
–Intestinal	208	52
Tumor Location		
–Antrum	240	60
–Corpus	100	25
–Fundus	40	10
–Gastroesophageal junction	20	5

Abbreviations: ECOG (Eastern Cooperative Oncology Group Performance Status).

**Table 2:** Initial Treatment Allocation After Diagnostic Laparoscopy.

Initial Diagnostic Laparoscopy Findings	*n*	%	Treatment Allocation
No evidence of peritoneal metastasis (PCI = 0)	320	80.0	Neoadjuvant chemotherapy
Peritoneal metastasis, PCI < 7	53	13.3	CRS + HIPEC
Peritoneal metastasis, PCI ≥ 7	22	5.5	PIPAC + systemic chemotherapy
Extensive disease/poor performance status	5	1.2	Best supportive care

Abbreviations: PCI, Peritoneal Cancer Index; CRS + HIPEC: Cytoreductive Surgery and Hyperthermic Intraperitoneal Chemotherapy; PIPAC: Pressurized Intraperitoneal Aerosol Chemotherapy.

**Table 3:** Comparison of Baseline Characteristics Between Patients Who Completed and Did Not Complete Neoadjuvant Chemotherapy.

Variable	Completed (*n* = 261)	Did Not Complete (*n* = 59)	*p*-Value
Age (years), mean ± SD	60.9 ± 10.8	63.1 ± 11.6	0.176
Male sex, *n* (%)	172 (65.9)	38 (64.4)	0.821
ECOG 0–1, *n* (%)	194 (74.3)	38 (64.4)	0.118
ECOG 2–3, *n* (%)	67 (25.7)	21 (35.6)	
Diffuse histology, *n* (%)	123 (47.1)	31 (52.5)	0.462
Intestinal histology, *n* (%)	138 (52.9)	28 (47.5)	
Tumor location—Antrum, *n* (%)	160 (61.3)	33 (55.9)	0.583
Corpus/Fundus/GEJ, *n* (%)	101 (38.7)	26 (44.1)	
Clinical T3–T4, *n* (%)	221 (84.7)	52 (88.1)	0.514
Clinical N+, *n* (%)	198 (75.9)	47 (79.7)	0.548

**Table 4:** Neoadjuvant Chemotherapy-Related Complications.

Complication	*n*	%
Grade 3–4 hematologic toxicity	39	15
Gastrointestinal intolerance	33	12.6
Partial obstruction	7	2.7
Minimal bleeding	5	1.9

**Table 5:** CRS plus HIPEC Outcomes (*n* = 70).

Complication	*N*	%
Anastomotic leakage	4	5.7
Postoperative infection	7	10.0
Late bowel obstruction	3	4.3
Hematological toxicity	4	5.7
Overall morbidity	18	25.7
Surgical mortality	2	2.9
Median follow-up (months)	–	30
One-year overall survival	–	70

**Table 6:** PIPAC Outcomes (*n* = 39).

Complication	*N*	%
Nausea and vomiting	6	15.4
Transient hematological toxicity	3	7.7
Minor intra-abdominal bleeding	1	2.6
Overall morbidity	10	25.6
Median follow-up (months)	–	22
One-year overall survival	–	50

**Table 7:** Palliative Surgery Outcomes (*n* = 39).

Parameter	Value
Median follow-up (months)	6
One-year overall survival (%)	15
Overall morbidity (%)	23.1

**Table 8:** Univariate Cox Regression Analysis for Overall Survival.

Variable	Hazard Ratio (HR)	95% CI	*p*-Value
Age (per 10-year increase)	1.12	0.95–1.33	0.176
Sex (Male vs. Female)	1.08	0.81–1.43	0.594
ECOG 2–3 vs. 0–1	1.78	1.23–2.57	0.002
Histology (Diffuse vs. Intestinal)	1.22	0.92–1.62	0.148
Tumor location (Non-antral vs. Antral)	1.10	0.82–1.47	0.521
Baseline PCI (per 1-point increase)	1.12	1.07–1.17	<0.001
Tumor size (>5 cm vs. ≤5 cm)	2.18	1.52–3.13	<0.001
T stage (T3–T4 vs. T1–T2)	3.41	2.15–5.42	<0.001
N stage (N+ vs. N0)	2.96	2.04–4.28	<0.001
TNM stage (III–IV vs. I–II)	3.85	2.47–6.02	<0.001
Histological grade (G3 vs. G1–2)	1.89	1.33–2.69	<0.001
Lymphovascular invasion (Present vs. Absent)	1.67	1.16–2.40	0.005

Abbreviations: HR, hazard ratio; CI, confidence interval.

**Table 9:** Full Multivariable Cox Proportional Hazards Regression Model for Overall Survival.

Variable	Adjusted HR	95% CI	*p*-Value
Age (per 10-year increase)	1.18	0.95–1.46	0.134
Sex (Male vs. Female)	1.09	0.81–1.47	0.561
ECOG 2–3 vs. 0–1	1.52	1.03–2.23	0.034
Histology (Diffuse vs. Intestinal)	1.27	0.94–1.72	0.118
Tumor location (Non-antral vs. Antral)	1.15	0.84–1.58	0.372
Baseline PCI (per 1-point increase)	1.09	1.03–1.15	0.002
Tumor size (>5 cm vs. ≤5 cm)	1.34	0.91–1.98	0.141
T stage (T3–T4 vs. T1–T2)	2.63	1.71–4.06	<0.001
N stage (N+ vs. N0)	2.21	1.52–3.23	<0.001
Histological grade (G3 vs. G1–2)	1.22	0.84–1.78	0.298
Lymphovascular invasion (Present vs. Absent)	1.18	0.81–1.72	0.388

Abbreviations: HR, hazard ratio; CI, confidence interval; ECOG, Eastern Cooperative Oncology Group; PCI, Peritoneal Cancer Index.

## Data Availability

The data that support the findings of this study are not publicly available due to privacy and ethical restrictions regarding patient confidentiality, but are available from the corresponding author upon reasonable request.
